# The pivotal roles of exosomes derived from endogenous immune cells and exogenous stem cells in myocardial repair after acute myocardial infarction

**DOI:** 10.7150/thno.53326

**Published:** 2021-01-01

**Authors:** Yu-Yan Xiong, Zhao-Ting Gong, Rui-Jie Tang, Yue-Jin Yang

**Affiliations:** State Key Laboratory of Cardiovascular Disease, Department of Cardiology, Fuwai Hospital, National Center for Cardiovascular Diseases, Chinese Academy of Medical Science and Peking Union Medical College, Beijing 100037, China

**Keywords:** Myocardial infarction, exosome, immunomodulation, immune cells, stem cells

## Abstract

Acute myocardial infarction (AMI) is one of the leading causes of mortality around the world, and the inflammatory response plays a pivotal role in the progress of myocardial necrosis and ventricular remodeling, dysfunction and heart failure after AMI. Therapies aimed at modulating immune response after AMI on a molecular and cellular basis are urgently needed. Exosomes are a type of extracellular vesicles which contain a large amount of biologically active substances, like lipids, nucleic acids, proteins and so on. Emerging evidence suggests key roles of exosomes in immune regulation post AMI. A variety of immune cells participate in the immunomodulation after AMI, working together to clean up necrotic tissue and repair damaged myocardium. Stem cell therapy for myocardial infarction has long been a research hotspot during the last two decades and exosomes secreted by stem cells are important active substances and have similar therapeutic effects of immunomodulation, anti-apoptosis, anti-fibrotic and angiogenesis to those of stem cells themselves. Therefore, in this review, we focus on the characteristics and roles of exosomes produced by both of endogenous immune cells and exogenous stem cells in myocardial repair through immunomodulation after AMI.

## Introduction

Acute myocardial infarction (AMI) has long been a major cause of death in coronary artery disease worldwide despite the improved medical care 1, 2. When blood supply is abruptly blocked in coronary artery, massive cardiomyocytes undergo necrotic process and an intense inflammatory response is then triggered to clear necrotic debris. In the early phase of inflammation response dominated by immune cells after AMI, the intensive pro-inflammatory cytokines and chemokines are released to outbreak inflammatory process to digest damaged cells and extracellular matrix (ECM) tissue. In the next several days, the inflammatory phase gradually switches to reparative phase including inflammation resolution, neovascularization and scar formation. The expansion of immune cells and excessive prolonged inflammation response contribute to ischemic cardiomyopathy, which makes targeting inflammation response after myocardial infarction (MI) a potential strategy to attenuate myocardial dysfunction and heart failure (HF) 3, 4.

Exosomes, secreted by cells to extracellular space via exocytosis, is a vital way of intercellular communication. Formed by a lipid bilayer of plasma membrane origin and having multifarious biological cargo contents such as lipids, proteins, and RNAs, exosomes are involved in numerous physiological processes including immune regulation 5. In recent years, their roles in immune regulation on a molecular and cellular basis have been gradually unveiled in the context of AMI 6, 7. Meanwhile, immune cells and stem cells, which are important cell therapy for AMI, have been confirmed as promising strategies for immunomodulation of AMI 8, 9. Therefore, in this review we will summarize the characteristics and biological function of exosomes and the roles of exosomes derived from immune cells and stem cells in cardiac repair through modulation of immune responses post MI.

## Exosomes: secreted vesicles for intercellular communications

Exosomes, a major subgroup of extracellular vesicles (EVs), generally range in size from 30 to 200 nm in diameter 10. They can be found in most body fluids including plasma, serum, saliva, amniotic fluid, breast milk, and urine 11, and they can be released by various cell types such as dendritic cells (DCs), mast cells, platelets 12, as well as mesenchymal stromal cells (MSCs) 13.

**Figure 1:**
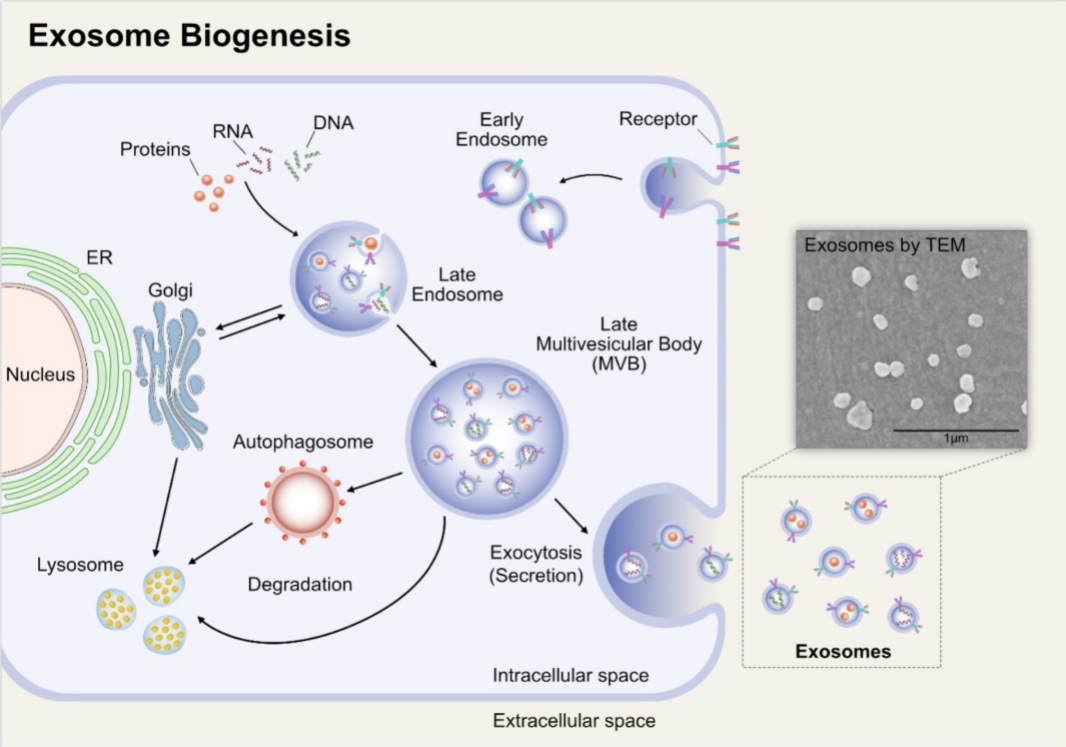
** Biogenesis of exosomes.** Early endosomes are generated by endocytosis of parent cell. It will then undergo the second invagination of the plasma membrane, thus forming ILVs, and the endosomes that enclose the ILVs are MVBs. MVBs can fuse with the plasma membrane and release the ILVs, namely exosomes. Adapted with permission from 15, Copyright (2019).

Exosomes will undergo double invagination of the plasma membrane. The first invagination is accompanied by endocytosis of parent cell, and then the early endosomes are generated in cytoplasma. Early endosomes can mature into late endosomes and finally multivesicular bodies (MVBs) or multivesicular endosomes. The MVBs will then undergo the second invagination of the plasma membrane, thus forming intraluminal vesicles (ILVs). There are two outcomes, to fuse with lysosomes or autophagosomes undergoing degradation or to fuse with the plasma membrane and release the ILVs, that is what we called exosomes 14 (**Figure 1**).

Exosomes have been confirmed to be vital carriers of unique cargo of lipids, proteins and RNAs, which are usually distinct from the parent cell of its origin 14, 16. It has been proposed that exosomes bind to the plasma membrane of recipient cells via specific receptors and are either internalized by micropinocytosis to fuse with the membrane to release its contents of lipids, proteins and RNAs 17, 18 or are internalized by distinct endocytosis. Because of these characteristics that they have, exosomes seem to be capable of acting as vehicles for drug delivery to convey its RNA and protein contents.

Multiple cell types including cardiomyocytes, endothelial cells, cardiac fibroblasts and immune cells work together to make the heart function properly. In response to distinct types of stress, different kinds of cardiac cells are able to secrete biological molecules to mediate intercellular communication in which exosome plays an essential role. For example, under ischemic conditions, miR-222 and miR-143 are abundant in exosomes derived from cardiomyocytes which stimulate the neovascularization following AMI 19. Endothelial cells, which are crucial for the establishment and maintenance of vascular integrity, could release exosomes that contain miR-214 to stimulate angiogenesis 20. Taken together, these data indicate the importance of exosomes in intercellular communications between different cell types.

## Two phases of inflammatory responses after AMI

**Figure 2:**
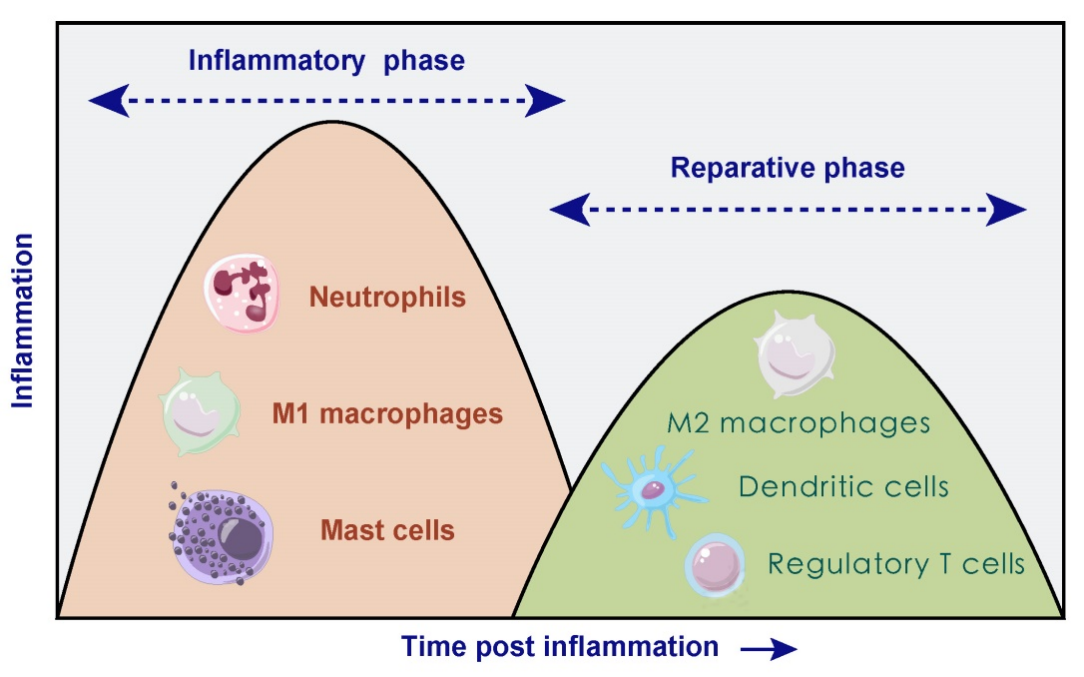
** Temporal two different phases of inflammatory process after AMI**. In inflammatory phase, neutrophils, M1 macrophages and mast cells dominated, accompanied with damaged cells and tissue digestion. During the following reparative phase, macrophages polarized towards anti-inflammatory type, and dendritic cells as well as regulatory T cells both participated in the resolution of inflammation.

Due to the necrosis of infarcted myocardium, vascular endothelial cell integrity and its barrier function are impaired accompanied with sudden massive loss of cardiomyocytes, facilitating the release of danger-associated molecular patterns (DAMPs) 21. DAMPs are cytoplasmic or nuclear components that can be released into the extracellular environment due to cell necrosis, including heat shock proteins, high mobility group box 1. It can activate the immune system thus triggering immune responses 22 via binding to cognate pattern recognition receptors containing toll-like receptor/interleukin 1 receptors (TLR/IL1R) and nucleotide-binding oligomerization domain-like receptors on surviving cardiomyocytes 23-28. In turn, receptor activation triggers intercellular crosstalk signal and results in the release of various pro-inflammatory mediators. Cardiomyocyte-released chemokines promote immune cell extravasation and recruitment through binding to the related chemokine receptors, and the up-regulation of pro-inflammatory cytokines [including tumor necrosis factor (TNF), interleukin 1β (IL1B), interleukin 6 (IL6)] promote adhesive interactions between leukocytes and endothelial cells, thus leading to large amounts of inflammatory cells transmigrating into infarcted myocardium 29. In the early stage of AMI, neutrophils are recruited to the infarct area within hours after cardiac injury, reaching a peak at day 1-3 and declining to normal level at day 5-7 30. Then M1 macrophages dominate and participate in the phagocytosis of necrotic tissue together with neutrophils. Necrotic or damaged cells and ECM tissue are then digested and cleared, followed by a reparative phase over the next several days. The transition to the reparative phase depends on the timely suppression of the inflammatory response, and anti-inflammatory monocyte subtypes, lymphocytes and anti-inflammatory macrophages may be involved in this period 31. During the reparative phase, neutrophils rapidly undergo cell death, inducing a M2 phenotype conversion in macrophages and secretion of anti-inflammatory and pro-fibrotic cytokines such as IL10 and transforming growth factor beta (TGFB) which suppress inflammation and promote tissue repair. The polarization of macrophages stimulates the production of vascular endothelial growth factor (VEGF) and TGFB and then promotes angiogenesis and ECM synthesis 32. Besides, bone marrow derived DCs infiltrate the necrotic myocardium, predominantly during the reparative phase 33, 34. It seems like the filtration of DCs after AMI can control macrophage homeostasis thus modulating the postinfarction healing process 34. In addition, T cells and mast cells both participate in immune response to varying degree (**Figure 2**).

The inflammatory process participates in clearing dead cells, facilitating scar formation whereas excessive or prolonged inflammation response leads to degradation of extracellular matrix, resulting in dilative remodeling and HF 35, which makes the process of immune response a novel target for the treatment of AMI and the prevention of HF.

## Immune cell-derived exosomes in immunomodulation after AMI

The immune system plays a vital role in pathogens defense, inflammation response, and wound repair. Immune cells predominantly participate in clearing out cell debris, inflammation resolution and healing process post AMI 34, 36. Emerging evidence has indicated that exosomes derived from immune cells are essential in carrying out these functions 37. Exosomes have been increasingly researched and applied to the salvage of ischemic myocardium, from which we can speculate that exosomes from immune cells might become potential alternatives for the treatment of AMI patients.

### Exosomes from macrophages

In the infarcted myocardium, two sequential sets of macrophages, namely M1 macrophage and M2 macrophage, dominate in two different phases of inflammatory process after AMI. In inflammatory phase, M1 macrophage, which is proinflammatory type, secretes massive pro-inflammatory mediators. In the reparative phase, M2 macrophage dominates in the infarcted myocardium and facilitates wound repair via myofibroblast activation, angiogenesis and ECM deposition.

In injured heart, miR-155 derived from activated cardiac macrophages could be transferred into cardiac fibroblasts, thus inhibiting proliferation of fibroblasts, enhancing inflammation with the upregulation of tumor necrosis factor alpha (TNFA), IL1B, and C-C Motif Chemokine Ligand 2 (CCL2), decreasing collagen production and promoting cardiac rupture via targeting Son of Sevenless gene 1 and Suppressor of Cytokine Signaling 1 38. Additionally, macrophages were also recipients of miR-155-enriched exosomes from endothelial cells, which further shifted the macrophage balance from anti-inflammatory M2 macrophages towards proinflammatory M1 macrophages 39. Further evidence confirmed that exosomes secreted by pro-inflammatory M1 macrophages exerted an anti-angiogenic effect and accelerated MI injury 40, which partly due to the highly expressed proinflammatory miR-155 contained in those exosomes and led to inhibition of angiogenesis and cardiac dysfunction. On the contrary, M2 macrophage-derived exosomes enhanced the viability of cardiomyocytes and reduced myocardial ischemia/reperfusion (I/R) injury *in vivo* mainly via highly expressed miR-148a 41. The elevation of miR-148a expression has also been proven to impair B cell tolerance via facilitating the survival of immature B cells by means of downregulating the expression of growth arrest and DNA-damage-inducible 45 alpha, phosphatase and tensin homolog (*Pten*) and BCL2-like 11 which encodes the pro-apoptotic factor Bim 42. Therefore, macrophages may be able to regulate immune responses by transferring miRNAs to B cells. Taken together, different contents including miR-155 and miR-148a derived from macrophages could effectively modulate immune response thus providing new targets for the treatment of AMI.

### Exosomes from DCs

DCs, pivotal antigen-presenting cells, are key to the immunological response with different functions participating in immunity 43-45. Emerging evidence confirmed that DCs were involved in the pathophysiological mechanisms of various cardiovascular diseases such as atherosclerosis, hypertension and HF 46, 47. In the infarcted myocardium, DCs were vital in recruiting and activating immune cells particularly macrophages and T cells, accompanied by a notably increase of inflammatory cytokines 48. Meanwhile, released EVs of DCs have been reported as an important way of mediating intercellular communication in immunity. Although the majority of studies of DC-derived exosomes focused on immunotherapy against various types of cancer, rising attention has been paid to the role of exosomes derived from DCs in AMI.

After AMI, DCs migrated to the infarction border zone and participated in the activation of lymphocytes and the initiation of immune responses 48, 49. Further study indicated that mice with DCs ablation showed enhanced and sustained expression of inflammatory cytokines (such as IL1B, IL18, and TNFA), prolonged ECM degradation and enhanced proinflammatory M1 macrophage recruitment after AMI 34. Injection of DCs to the infarcted mice induced a systemic activation of MI-specific regulatory T cells (Tregs) and facilitated an M2 macrophage shift, resulting in better wound healing and preserved left ventricular systolic function 50. Furthermore, the injection of exosomes secreted from DCs could directly activate CD4^+^ T cells through Th1 signaling pathway. Despite that the inflammatory cytokines were upregulated; the injection of exosomes derived from DCs effectively improved the cardiac function of mice post-MI 51. Considering that the activated CD4^+^ T cells could facilitate wound healing of the myocardium after AMI 36, it is reasonable to speculate that exosomes from DCs might activate CD4^+^ T cells to exert cardioprotective effects after infarction. But the experiments of Cai *et al* demonstrated that miR-142-3p enriched in exosomes derived from activated CD4^+^ T cells (CD4-activated Exos) targeted and inhibited the expression of Adenomatous Polyposis Coli, contributing to the activation of WNT signaling pathway and activation of cardiac fibroblast, thus evoking pro-fibrotic effects of cardiac fibroblasts. And the delivery of CD4-activated Exos into the heart aggravated cardiac fibrosis and caused post-MI dysfunction 52. Therefore, the cardioprotective effects of exosomes secreted from DCs deserve further research.

### Exosomes from Tregs

Tregs are a specific subset of T lymphocytes with immunosuppressive effects, which counts 5-10% of CD4^+^ T cells in human peripheral blood 53. They are essential in enhancing the polarization of anti-inflammatory M2 macrophages 54, 55, elevating the levels of anti-inflammatory cytokines including IL10, IL4, IL13 and reducing the secretion of pro-inflammatory cytokines 54, 56. It has been confirmed that exosomes derived from Tregs could transfer miRNAs especially miR-150-5p and miR-142-3p to DCs accompanied with reduced immune reactions 57. MiR-150 was pivotal in attenuating immune responses of DCs and protecting cardiomyocytes from cell death under conditions of hypoxia 58. Additionally, miR-150 was a critical passive regulator of monocyte cell migration and suppressed pro-inflammatory cytokines production, leading to cardioprotective effects 59. Upregulation of miR-142-3p resulted in shrinking I/R damage-triggered infarct size, strengthening cardiac function and guarding against cardiomyocyte apoptosis 60. Meanwhile, exosomes from Tregs cells could transfer Let-7d to T helper 1 (Th1) cells and suppressed proliferation of Th1 cells and secretion of pro-inflammatory cytokines 61. Infiltration of Th1 cells led to cardiac fibroblasts activation, then cardiac fibroblasts transformed into myofibroblasts via integrin α4. In addition, Th1 cells induced *Tgfb* expression in myofibroblasts, which facilitated the formation of fibrillary ECM in the myocardium thus promoting cardiac fibrosis 62. Based on the above research, Tregs-derived exosomes may exhibit its cardioprotective effects by interacting with other immune cells.

### Exosomes from mast cells

Mast cells have been directly linked to atherosclerotic plaque rupture which results in acute thrombotic occlusion of the coronary artery and thus leading to AMI 63. The inhibition of chymase secreted by mast cells led to reduced *Tgfb* expression accompanied with reduced myocardial fibrosis and cardiac dysfunction 64. Interestingly, tryptase secreted by mast cells contributed to the angiogenesis and promoted the healing process in the infarcted myocardium 65. To summarize, mast cells participate not only in the generation of MI but also in the reparative process via its diverse mediators.

**Figure 3:**
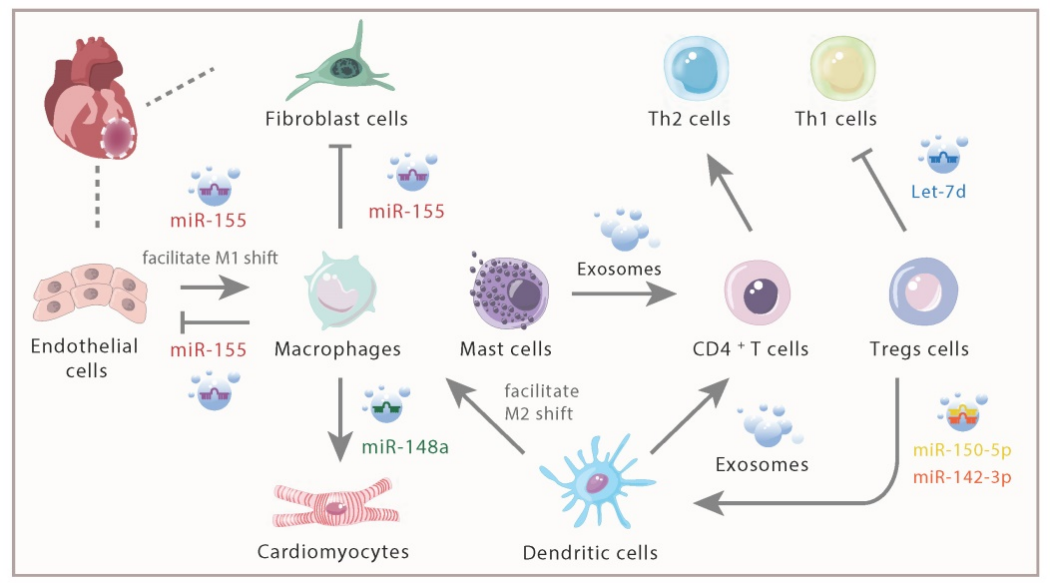
** Intercellular communication of immune cells and cardiac inherent cells via exosomes and its contents**. In response to AMI, distinct immune cells infiltrated within the infarcted myocardium. Exosomes, with various derivation and different contents, played different roles in inflammation response.

A research confirmed that mast cells can exhibit its inflammatory and immunoregulatory functions via exosomes in addition to cell-to-cell contacts and cytokines release 66. The data also indicated that exosomes derived from mast cells were capable of activating B and T lymphocytes, suggesting that exosomes derived from mast cells may participate in the development and the amplification of both the specific and nonspecific inflammatory responses. Exosomes derived from mast cells also could partially promote the proliferation of CD4+ T cells and dramatically enhance the differentiation of naïve CD4+ T cells to Th2 cells 67, presenting an immunoregulatory effect (**Figure 3**).

### Stem cell-derived exosomes in immunomodulation after AMI

Stem cell transplantation has been recognized as a highly attractive option for the treatment of infarcted myocardium while increasing evidence suggests that its cardioprotective effects mainly depend on paracrine way. Therefore, stem cell-derived exosomes transplantation is considered to be a promising treatment for MI. Besides, compared with endogenous immune cell derived exosomes, exogenous stem cells-derived exosomes are also inseparable from immune regulation. In this part, we mainly focus on exosomes derived from MSCs, cardiac progenitor cells (CPCs) and cardiosphere cells (CDCs), and the mechanisms related to their cardioprotective functions are listed in **Table 1**.

### MSC derived exosomes

MSCs are a group of adult stem cells with self-renewal and differentiation abilities and also immunomodulatory properties, and have been widely used in tissue repair and regeneration 68. They express CD73, CD90, and CD105, and don't express CD45, CD34, CD14, CD19, CD11b, and human leukocyte antigen DR isotype 69. Due to their characteristics of easy isolation, convenient acquisition and low immunogenicity, they have become the most promising stem cell type in the treatment of AMI. According to their original sources, MSCs can be divided into bone marrow derived MSCs (BMMSCs), adipose tissue derived MSCs (ADSCs), umbilical cord derived MSCs (ucMSCs), and so on. The view that main benefits of MSC therapy are derived from secreted factors acting on neighboring cells through paracrine way has already become a widely accepted point 70. As indispensable paracrine substances, exosomes derived from MSCs have proven to show similar effects as MSCs, including anti-apoptosis, promoting angiogenesis, and also immunomodulation in the treatment of AMI.

### BMMSC-derived exosomes

Many studies have found that BMMSC-derived exosomes (BMMSC-Exos) can regulate the local inflammatory cytokines in infarcted myocardium. The injection of BMMSC-Exos could greatly repress inflammatory cytokines including IL1B, IL6 and TNFA which were induced by AMI, as well as targeting pro-apoptotic proteins like FASL and PTEN to alleviate MI mainly through miR-25 71. Further studies confirmed that BMMSC-Exos could promote the polarization of M1 macrophages to the M2 macrophages both* in vivo* and *in vitro*, thereby alleviating inflammation response. The miRNA sequencing and bioinformatics analysis of BMMSC-Exos indicated that miR-182 was a potential candidate mediator for modifying macrophage polarization via targeting *Tlr4* 72. The immunoregulatory effects of BMMSC-Exos on macrophages can be further enhanced by artificial means including drug pretreatment and gene modification. Xu *et al* pretreated BMMSCs with low-dose lipopolysaccharide (LPS) and collected the exosomes (L-Exos). L-Exos had superior therapeutic effects on mediating macrophage polarization and further alleviated post-MI inflammation and cardiomyocyte apoptosis 73. Exosomes derived from BMMSCs pretreated with atorvastatin had an elevated level of lncRNA H19 and reduced the inflammatory cytokines with markedly promoting angiogenesis, minimizing infarct size and improving ventricular function post MI 74. Furthermore, engineered exosomes with ischemic myocardium targeting peptide exerted more accumulation in ischemic myocardium and enhanced therapeutic effects on attenuating inflammation and cardiomyocytes apoptosis 75. Meanwhile, BMMSC-Exos impaired T-cell function via inhibiting its proliferation, and also restrained the inflammation response as well as improved cardiac function 76. Until now, there is a lack of research focusing on BMMSC-Exos regulating other immune cells, but studies have shown that DCs could regulate macrophage polarization and the Tregs, and also participated in presenting antigens to T cells to activate CD4^+^ T cells 50, 51. As the regulation of BMMSC on DCs has been confirmed by many experiments 77, the regulation of DCs by BMMSC-Exos will further improve the understanding of the mechanism of exosomes to regulate immune response after AMI.

### ADSCs-derived exosomes

The concentration of MSCs in adipose tissue is notably higher than in bone marrow (1% versus 0.01%) and other sources 78. Compared to the bone marrow, harvesting MSCs from adipose tissue is less invasive and has no ethical limitations 79. Similar to BMMSCs, ADSCs can differentiate into ectodermal, endodermal as well as the mesodermal lineage, and also exhibit immunomodulatory characteristics 80. During the inflammatory phase, M1 macrophage predominant in the infarcted myocardium and proinflammatory cytokines including IL6, IL1B, interferon γ (IFNG) and TNFA are elevated. When treated with exosomes derived from miR-126-overexpressing ADSCs, inflammatory cytokines expression and cardiac fibrosis were notably decreased 81. Further studies indicated that the immunomodulatory effects of ADSCs-derived exosomes might be associated with macrophage polarization. Deng *et al.* confirmed that ADSCs-derived exosomes treatment effectively promoted macrophage polarization to M2 type, which inhibited inflammatory responses and attenuated myocardial fibrosis by suppressing *Nfkb* and *Tgfb1* expression 82. In addition, exosomes released from ADSCs under stimulation with IFNG and TNFA showed strengthened immunosuppressive and anti-inflammatory effects 83.

### HucMSCs derived exosomes

Compared with other MSCs, hucMSCs have the characteristics of low cost, low invasiveness, easy isolation, high cell content, high gene transfection efficiency and low immunogenicity, which arouse interests of scientists in tissue repair 84. Exosomes derived from hucMSC (hucMSC-Exos) are promising new treatment options for AMI. MiR-19a could suppress apoptosis of myocardial cells 85 and was detected to be lower in myocardial tissues of AMI compared to normal tissues, while hucMSC-Exos significantly increased the release of miR-19a and attenuated ischemic injury with decreased expression of inflammatory cytokines 86. Additionally, Shi* et al.* found that on day 2 after AMI, the pro-inflammatory factors were downregulated and anti-inflammatory factors were upregulated in the infarcted myocardial tissue in rats when treated with hucMSC-Exos 87, confirming that hucMSC-Exos were involved in regulating the local immune microenvironment after AMI. Since miR-181a has been confirmed to be associated with inflammatory-related disease 88 and was involved in Tregs activation 89, Wei *et al.* utilized exosomes derived from miR-181a overexpressing hucMSCs to alleviate the cardiac injury post I/R and they found the exosome treatment created an anti-inflammatory environment, and also increased Tregs polarization 90 which were capable of promoting the conversion of the pro-inflammatory phase to the pro-reparative phase and participating in wound healing through modulating macrophage differentiation 91. When encapsulated in functional peptide hydrogels, hucMSC-Exos exhibited increased retention within the myocardium and showed better immunomodulatory and cardioprotective effects 92. Based on the current limited experimental evidence, we believe that hucMSC-Exos also have strong immunoregulatory abilities, and they are quite promising in the treatment of AMI and deserve further research.

### CPC derived exosomes

Progenitor cell is a kind of stem cell that is distinct from embryonic stem cell (ESC) for its predetermined differentiation fate and its limited potential of self-renewal as well as differentiation into other cell types 93. CPCs can differentiate into cardiomyocytes and endothelial cells 94. Although CPCs are considered as quiescent cells in physiological conditions, it is suggested that they can be activated in injury and may differentiate into cardiac cells 95. The translational relevance of CPCs in cardiac therapy has been proven in several studies, and promising results have been obtained in preclinical studies and clinical trials 96.

Studies have pointed out that CPCs have a strong ability to suppress immunity, and this effect is mainly mediated via paracrine way. When co-culture with CPCs, the proliferation of T cell was notably reduced accompanied with strong downregulation of* IFNG* and *TNFA*, and EVs play an important role in this process 97. *In vivo*, soluble junctional adhesion molecule-A in the conditioned medium from CPCs reduced neutrophils infiltration after AMI and reduced tissue damage by preventing excessive inflammation 98. Proteomics analysis demonstrated that pregnancy-related plasma protein A is one of the highest contents of CPC-derived exosomes in comparison to BMMSC-Exo, while the injection of CPC-derived exosomes exhibited less CD68+ macrophage infiltration, reflecting its immunomodulatory effects 99.

### CDC derived exosomes

CDCs are a group of CPCs that have the ability to motivate endogenous mechanisms of cardiac repair and attenuate adverse ventricular remodeling 100, and also have been proven to improve cardiac function in a variety of heart diseases 101. CDC-derived exosomes (CDC-Exos) could also mitigate the myocardium damage caused by AMI, having the ability to relieve oxidative stress, reduce cell apoptosis and adverse ventricular remodeling, and facilitate angiogenesis after MI 102-105. Meanwhile, several experiments have demonstrated the immunoregulatory effects of CDC-Exos within the infarcted myocardium. Administration of CDC-Exos modified the polarization to M2 macrophage phenotype and enhanced the endogenous phagocytic capacity of macrophage, thus promoting the clearance of necrotic cell debris and also relieving excessive proinflammatory stress within the infarcted heart, facilitating the recovery of cardiac function after AMI. In this process, the highly expressed miR-181b in CDC-Exos which acted as a significant candidate mediator of CDC-induced macrophage polarization exerted its downstream functions by targeting protein kinase C delta (*Prkcd*) 106. Moreover, the high abundance of Y RNA fragment in EVs derived from CDCs could target macrophages and then enhanced IL10 protein secretion which could stimulate monocytes and prevent excessive inflammatory reactions 107, 108. CDC-derived EVs were also found to be involved in polarizing M1 macrophage to a proangiogenic phenotype through the upregulation of arginase 1 109. Besides, engineered CDCs with cardiomyocyte specific peptide endowed the exosomes with better targeting and retention ability and also superior immunoregulatory effects 110.

ESCs and induced pluripotent stem cells are regarded as highly attractive methods for the treatment of AMI. Their exosomes also have similar therapeutic effects 105, 111, 112, but specific studies focused on immunomodulation are still scarce.

## Conclusions and Perspectives

The inflammatory response mediated by various immune cells as well as inflammatory factors play vital roles in the process of myocardial necrosis and repair after AMI. Excessive inflammation response or improper suppression of inflammation may both affect the myocardial repair, leading to ventricular remodeling, and deterioration of heart function and development of HF after AMI. As important mediators of cell communication, exosomes are crucial in regulating immune cells and immune responses after AMI, facilitating the reparative process of infarcted myocardium, preserving ventricular function via the communication between lymphocytes or between lymphocytes and cardiac intrinsic cells. Systemic deliveries of exosomes derived from immune cells have gradually been recognized as a potent new therapeutic option for the treatment of MI-damaged myocardium. Moreover, stem cell-derived exosomes also have powerful immunomodulatory and inflammation inhibitory effects. They can act by directly targeting inflammatory cells or regulating inflammatory cytokines. Therefore, exosomes from both endogenous immune cells and exogenous stem cells are potential therapeutic strategies, which are promising for the treatment of AMI and worthy of further research for improving the prognosis of patients with AMI. Additionally, exosomes hold great potential of being therapeutic drug delivery vesicles due to its natural material transportation properties and excellent biocompatibility characteristics. Well-designed engineered exosomes may provide opportunities to enhance its therapeutic effects, making it promising and inspiring tools for clinical use 113. For example, conjugating the exosomes derived from CDCs with cardiac homing peptide effectively enhanced its therapeutic efficacy in cardiac repair and decreased the effective dose of intravenously delivery 114.

Derived from various cells, the heterogeneity of exosome sizes and contents is capable of reflecting the state and types of origin, making exosomes possible biomarkers for disease diagnostics 115. For example, exosomes containing miR-24 and miR-210 changed significantly correlated well with cTNI levels in patients undergoing coronary artery bypass grafting surgery, suggesting the potential role for exosomes as new biomarkers of myocardial injury 116. Circulating exosomes enriched in p53-responsive miRNAs including miR-34a, miR-192 and miR-194 have also been identified as prognostic biomarkers of MI 117. Although researches related to the application of exosomes derived from immune cells in cardiovascular diseases are scarce for now, circulating EVs derived from immune cells can act as biomarkers of other inflammation related diseases including chronic hepatitis C and nonalcoholic fatty liver 118. It is worthy of expecting that the identification of novel biomarkers from immune cell-derived exosomes will grow rapidly.

In conclusion, exosomes are emerging as important mediators of intercellular communication and exosomes derived from immune cells and stem cells are pivotal therapeutic tools in the treatment of AMI. Moreover, advanced modification strategies and detection methods in exosomes will provide us with great tools as therapeutic interventions and biomarkers for AMI. Considering the potential of being new generation of bio-nano drugs, exosomes have advantages in the field of cell-free therapy for cardiac repair post AMI as well as other diseases, and might produce enormous social and economic benefits.

## Figures and Tables

**Table 1:** Summarization of the derivation, effective components, mechanisms and biological effects of stem cells-derived exosomes in different pathological status.

Derivation of exosome	Effective components	Mechanisms	Biological effects	Pathological status	Reference
BMMSCs	miR-25-3p↑	miR-25-3p/*Ezh2*/*Socs3*	inflammation↓ apoptosis↓	MI	71
BMMSCs	miR-185↑	miR-185/*Socs2*	inflammation infiltration↓ apoptosis↓ ventricular remolding↓	MI	119
BMMSCs	miR-125b↑	miR-125b/*Sirt7*	IL1B, IL6, and TNFA↓ apoptosis↓	I/R	120
BMMSCs	miR-182↑	miR-182/*Tlr4*	M2 macrophages polarization↑	I/R	72
BMMSCs	LncRNA H19↑	LncRNA H19/miR-675/*Vegf* and *Icam1*	inflammation↓ angiogenesis↑ cardiomyocyte apoptosis↓ infarct size↓ cardiac function↑	MI	74
BMMSCs	LPS pre-conditioning	NFKB signaling pathway AKT1/AKT2 signaling pathway	M2 macrophages polarization↑ M1 macrophages polarization↓ inflammation↓	MI	73
BMMSCs	ischemic myocardium-targeting peptide↑	Not investigated	inflammation↓ apoptosis↓ fibrosis↓ vasculogenesis↑ cardiac function↑	MI	75
BMMSCs	indoleamine 2,3-dioxygenase↑	Not investigated	regulatory T-cells↑ CD8+ T-cells↓ pro-inflammatory cytokines↓ anti-inflammatory cytokines↑ allograft-targeting immune responses↓ cardiac allograft function↑	heart transplants	121
BMMSCs	miR-21↓ miR-15↓	Not investigated	inflammation↓ cardiac fibrosis↓ cardiac function↑ apoptosis↓ cell proliferation↑	AMI	122
BMMSCs	Not investigated	Not investigated	inflammation↓ neovascularization↑	AMI	76
BMMSCs	Not investigated	JAK2-STAT6	inflammatory cells infiltration↓ pro-inflammatory macrophages↓ cardiac function↑ cardiac dilation↓ cardiomyocytes apoptosis↓	dilated cardiomyopathy	123
ADMSCs	Hypothermia combination	PI3K/AKT/GSK3B p-m-TOR	TNFA and IL6↓ IL10↑ oxidative stress↓ apoptosis↓	I/R	124
ADMSCs	Not investigated	S1P/SK1/S1PR1 signaling	inflammatory response↓ M2 macrophages polarization↑ cardiac fibrosis↓ apoptosis↓	AMI	82
ADMSCs	miR-126↑	Not investigated	inflammation↓ apoptosis↓ fibrosis↓ angiogenesis↑	AMI	81
ADMSCs	Not investigated	Not investigated	M2 macrophages polarization↑	Pre-activated with inflammatory factors	83
hucMSCs	miR-181a ↑	Not investigated Not investigated	TNFA and IL6↓ IL10↑	I/R	90
hucMSCs	Encapsulated by hydrogel	Not investigated	inflammation↓ apoptosis↓ fibrosis↓ angiogenesis↑	AMI	92
MSCs	LncRNA KLF3-AS1	LncRNA KLF3-AS1/miR-138-5p/Sirt1	IL1B and IL18↓ cell apoptosis↓ pyroptosis↓	AMI	125
MSCs	Not investigated	PI3K/AKT Pathway	neutrophil infiltration ↓ macrophage infiltration↓ oxidative stress↓ adverse remodeling↓	I/R	126
CPCs	PAPPA↑	IGF1/AKT and ERK1/2	CD68+ macrophages↓ cardiomyocytes apoptosis↓ cardiac function↑	AMI	99
CDCs	miR-181b↑	miR-181b/ *Prkcd*	CD68+ macrophage within infarcted tissue↓ Modify polarization of macrophage	I/R	106
CDCs (EV)	Y RNA fragment	Not investigated	IL10↑ Infarct size↓	I/R	107
CDCs	Engineered with cardiomyocyte specific peptide	Not investigated	cardiac inflammation↓ fibrosis↓ cardiomyocyte apoptosis↓ cardiac retention↑	I/R	110
ESC	Not investigated	Not investigated	M2 macrophages↑ anti-inflammatory cytokine↑ cardiac remodeling↓	Doxorubicin-Induced Cardiomyopathy	127

miR: miRNA; EZH2: enhancer of zest homologue 2; SOCS: suppressor of cytokine signaling; SIRT7: sirtuin-7; IL1B: interleukin 1 beta; IL6: interleukin 6; TNFA: tumor necrosis factor alpha; TLR4: toll-like receptors 4; LncRNA H19: long non-coding rna h19; VEGF: vascular endothelial growth factor; ICAM1: intercellular cell adhesion molecule-1; LPS: lipopolysaccharide; NFKB: nuclear factor kappa-b; JAK2:Janus kinase 2; STAT6: signal transducer and activator of transcription 6; PI3K: phosphoinositide 3-kinase; GSK3B: glycogen synthase kinase 3β; p-m-TOR/p-AMKP: phosphorylate-mammalian target of rapamycin/ phosphorylate-adenosine 5'-monophosphate -activated protein kinase; IL10: interleukin 10; S1P/SK1/S1PR1: sphingosine 1-phosphate/sphingosine kinase 1/ sphingosine-1-phosphate receptor 1; Sirt1: sirtuin-1; IL18: interleukin 18; PAPPA: pregnancy-associated plasma protein a; IGF1: insulin-like growth factors-1; ERK1/2: extracellular regulated protein kinases 1/2; PRKCD: protein kinase c delta.

## Abbreviations

AMI: acute myocardial infarction
ECM: extracellular matrix
HF: heart failure
MI: myocardial infarction
EV: extracellular vesicles
DC: dendritic cell
MSC: mesenchymal stromal cell
MVB: multivesicular body
ILV: intraluminal vesicle
DAMP: danger-associated molecular pattern
TLR: toll-like receptor
IL1R: interleukin-1 receptor
IL1B: interleukin 1β
TNF: tumor necrosis factor
TNFA: tumor necrosis factor alpha
IL: interleukin
TGF: transforming growth factor
TGFB: transforming growth factor beta
VEGF: vascular endothelial growth factor
CCL2: c-c motif chemokine ligand 2
I/R: ischemia/reperfusion
PTEN: phosphatase and tensin homolog
IFNG: interferon-γ
Treg: regulatory T cell
Th1: T helper 1
CD4-activated Exos: exosomes derived from activated CD4^+^ T cells
CPC: cardiac progenitor cell
CDC: cardiosphere cell
ESC: embryonic stem cell
BMMSC: bone marrow derived MSCs
ADSC: adipose tissue derived MSCs
ucMSC: umbilical cord derived MSCs
BMMSC-Exo: BMMSC-derived exosomes
LPS: lipopolysaccharide
hucMSC-Exos: hucMSC-derived exosomes
CDC-Exos: CDC-derived exosomes
PRKCD: protein kinase c delta
